# Runx1-Stat3-Tgfb3 signaling network regulating the anterior palatal development

**DOI:** 10.1038/s41598-018-29681-3

**Published:** 2018-07-25

**Authors:** Safiye E. Sarper, Hiroshi Kurosaka, Toshihiro Inubushi, Hitomi Ono Minagi, Koh-ichi Kuremoto, Takayoshi Sakai, Ichiro Taniuchi, Takashi Yamashiro

**Affiliations:** 1 Department of Orthodontics and Dentofacial Orthopedics, Osaka University Graduate School of Dentistry, Osaka, Japan; 2 Department of Oral-facial Disorders, Osaka University Graduate School of Dentistry, Osaka, Japan; 3 Department of Advanced Prosthodontics, Graduate School of Biomedical & Health Sciences, Hiroshima University, Hiroshima, Japan; 4 Laboratory for Transcriptional Regulation, RIKEN Research Center for Allergy and Immunology, Yokohama, Japan

## Abstract

*Runx1* deficiency results in an anteriorly specific cleft palate at the boundary between the primary and secondary palates and in the first rugae area of the secondary palate in mice. However, the cellular and molecular pathogenesis underlying such regional specificity remain unknown. In this study, *Runx1* epithelial-specific deletion led to the failed disintegration of the contacting palatal epithelium and markedly downregulated *Tgfb3* expression in the primary palate and nasal septum. In culture, TGFB3 protein rescued the clefting of the mutant. Furthermore, Stat3 phosphorylation was disturbed in the corresponding cleft regions in *Runx1* mutants. The Stat3 function was manifested by palatal fusion defects in culture following Stat3 inhibitor treatment with significant downregulation of *Tgfb3*. Tgfb3 is therefore a critical target of Runx1 signaling, and this signaling axis could be mediated by Stat3 activation. Interestingly, the expression of *Socs3*, an inhibitor of Stat3, was specific in the primary palate and upregulated by *Runx1* deficiency. Thus, the involvement of Socs3 in Runx1-Tgfb3 signaling might explain, at least in part, the anteriorly specific downregulation of *Tgfb3* expression and Stat3 activity in *Runx1* mutants. This is the first study to show that the novel Runx1-Stat3-Tgfb3 axis is essential in anterior palatogenesis.

## Introduction

Cleft palate (CP) represents one of the major congenital craniofacial birth defects^1,2^. This condition demonstrates anatomical impairments with various combinations of the defects involving the soft and hard palates, the nasal septum and the alveolar ridge. Palatogenesis requires several developmental steps involving palatal shelf growth and elevation, as well as fusion of the palatal shelves. The palatal fusion begins at the midline of the future secondary palate following bilateral outgrowth of the maxillary process. Thereafter, the anterior secondary palate fuses to the primary palate and the dorsal portions of the secondary palate fuse with the nasal septum. Following contact, the intervening epithelium between the abutted shelves merges to form the epithelium seam that must be removed to provide mesenchymal continuity throughout the fused palate^3,4^. Fusion is crucial for the correct formation of the palate, and its defect can lead to CP. Although recent studies using genetically modified mice and human genetics studies have identified numerous genetic and environmental etiology for cleft palate, the etiology that explains the diversity in morphology in cleft palate has remained largely unknown.

We previously demonstrated that Runx1 participates in the pathogenesis of cleft palate using *Runx1*-null mutants carrying a *Gata1* promoter-driven *Runx1* transgene *(Gata1-Runx1*/*Runx1*^−/−^)^5^. Runx1 is a member of the Runx family of genes that encode transcription factors that play various important roles in embryogenesis^6^ and is a key molecule of hematopoiesis-causative genes in the development of leukemia and other hematopoietic disorders^7,8^. Runx1 is also involved in cancer development^9,10^. Indeed, families with children diagnosed with acute lymphoblastic leukemia reported a family history of clefts more often than control families^11^, and adult individuals who survived cancer reported a family history of cleft palate more often than controls^12^. Microdeletion encompassing *RUNX1* causes Braddock–Carey syndrome, which is characterized by distinct patterns of anomalies, including thrombocytopenia and cleft palate^13^.

Although *Runx1* is expressed in the fusing epithelium of the developing palatal shelf throughout the AP axis, it is of interest that *Runx1* mutants develop a localized anterior palatal clefting due to failed fusion between the primary and secondary palates and at the anterior-most part of the secondary palate corresponding to the 1st rugae^5^. This finding indicates that Runx1 is involved in a different regulatory mechanism for the palatal fusion along the anterior-posterior (AP) axis. Several studies have also revealed such different regulatory mechanism of palatogenesis along the AP axis of the palate^14,15^. Some transcription factors and signaling molecules, such as *Msx1*, *Bmp4*, *Shh*, *Fgf10*, *Fgf7*, and *Shox2* are recognized as anterior-specific^14,15^. Spatial distribution of such genes within the palate, together with the palatal phenotypes in their null mutant, clearly highlights the importance of regionally specific regulatory mechanism at the molecular level. However, the majority of experiments have focused on the palatal fusion of the secondary palate, and there is less understanding of the mechanisms in the anterior region between the primary and secondary palate and in the first rugae area of the second palate. Indeed, how Runx1 is involved in anterior-specific clefting has not been investigated.

In mouse skin cancer formation and maintenance, signal transducer and activator of transcription 3 (Stat3) is activated by Runx1 signaling as a tumor promoter^16,17^. The JAK/STAT pathway is the principal signaling mechanism for a wide array of cytokines and growth factors^18,19^. Upon activation, phosphorylated Stats dimerize and translocate to the nucleus, where they modulate the expression of target genes. It has been established that Stat3 is activated in a number of epithelial cancers^19^, in immune response, and also in response to various environmental signals^20^. Heterozygous loss-of-function mutations in *STAT3* lead to the primary immune deficiency Hyper-IgE syndrome and interestingly the cleft palate is observed in this syndrome^21^. However, little is known about the possible mechanisms underlying involvement of Stat3 in the pathogenesis of the cleft palate.

*Runx1* deficiency results in an anteriorly specific cleft palate at the boundary between the primary and secondary palates and in the first rugae area of the secondary palate in mice^5^. However, the cellular and molecular pathogenesis underlying such regional specific cleft palate remain unknown. In this study, using epithelial-specific *Runx1* deletion mice, we demonstrate that Tgfb3 is an essential target of Runx1 signaling in anterior-specific disintegration of the fusing palatal epithelium and that the site-specific downregulation of Stat3 phosphorylation plays a central role in downregulating the *Tgfb3* expression and manifesting palatal clefting upon *Runx1* loss. We also show that the pharmaceutical inhibition of Stat3 signaling disturbs the expression of *Tgfb3* as well as of *Runx1*, leading to failure of palatal fusion. In addition, we show that the involvement of Socs3 in Runx1-Tgfb3 signaling explain, at least in part, the anteriorly specific downregulation of *Tgfb3* expression and decreased Stat3 activity in *Runx1* mutants. Together, we demonstrate that Stat3-mediating Runx1-Tgfb3 axis is a novel regulatory pathway that regulates the palatal fusion in the anterior regions.

## Results

### Palatal phenotypes in Runx1 mutants

We previously reported that *Gata1-Runx1*/*Runx1*^−/−^ mice exhibit anterior clefting between the primary and secondary palates and in the first rugae region of the secondary palate and that *Runx1* is specifically expressed in the fusing epithelium, but the mechanisms underlying this phenotype remained largely unknown^5^. Since *Gata1-Runx1*/*Runx1*^−/−^ mice died within a few hours after birth, it was possible that the cleft palate phenotype was simply a consequence of delayed development. To extend the finding and examine how Runx1 regulates the anterior-specific palatal fusion in detail, we used epithelial-specific *Runx1* deletion mice (*K14-Cre/Runx1*^fl/fl^).

We also confirmed the efficiency of *K14-Cre* recombination using Rosa26R reporter mice at E15.0. β-gal staining was performed in the *K14-Cre;R26R* mice, and we confirmed that Cre recombination had been successfully performed in embryonic mouse in anterior palatogenesis. β-gal-positive cells were intense at the epithelium overlying the palatal process of the secondary palate and the primary palate (Fig. S1). The positive cells were evident in the contacting (B–E) and fused epithelium (F–H); however, no positive cells were detected in the mesenchyme underlying the palatal epithelium (Fig. S1).

**Figure 1:**
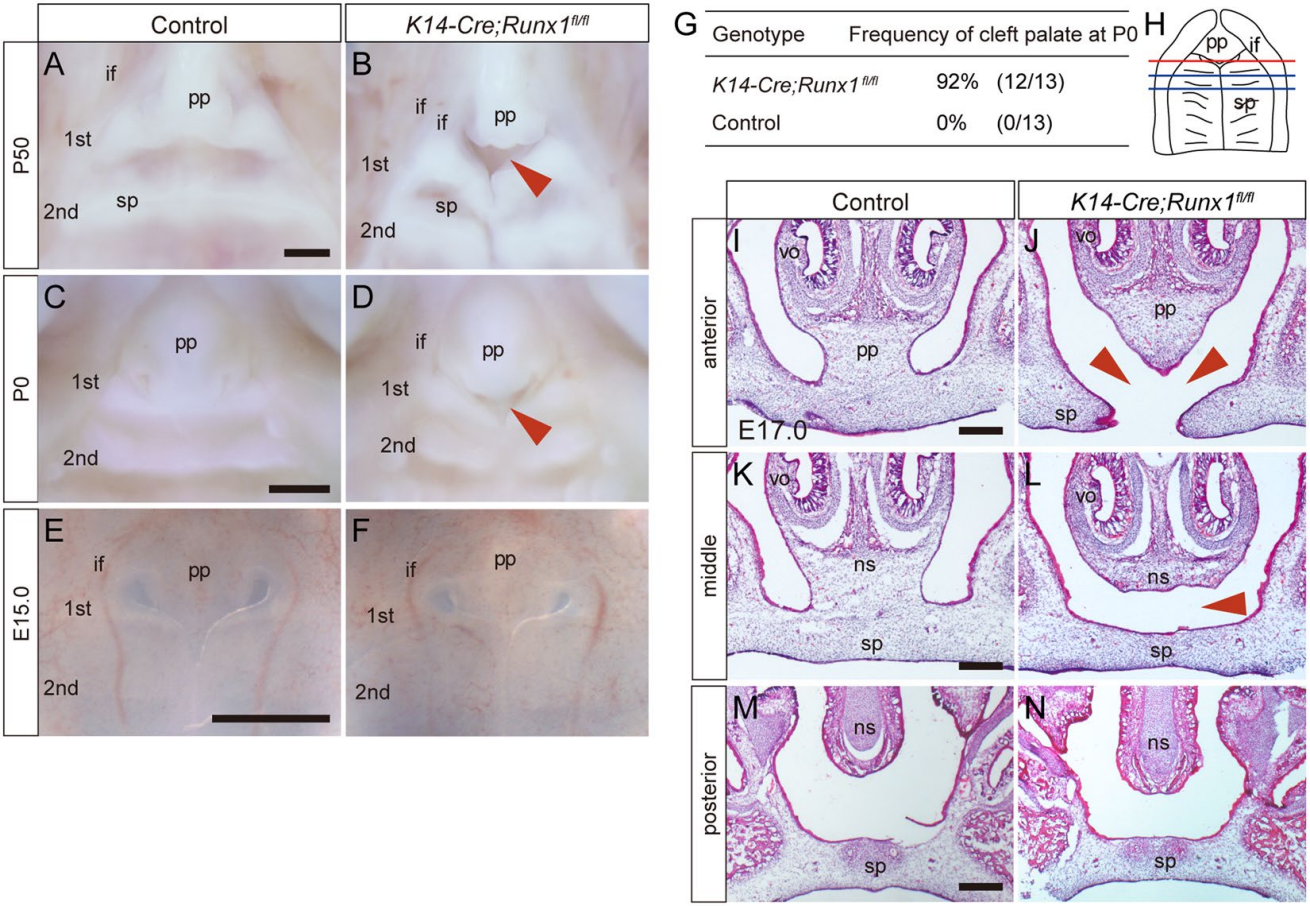
Palatal phenotypes of *K14-Cre/Runx1*^*fl/fl*^ mice. (**A**–**F**) Occlusal views of control and *K14-Cre/Runx1*^*fl/fl*^ mouse palate. An anterior cleft palate was evident at the boundary between the primary and secondary palate and at the first rugae area of the secondary palate in the *Runx1* mutant palate at both P50 and P0 (B,D, red arrowheads). Palatal phonotype was not evident at E15.0 (**E**,**F**). Scale bar: 1000 μm, P50; 500 μm, P0 and E15.0. (**G**) Frequency of anterior cleft of control and *Runx1* mutant mice at P0. (**H**) Diagram of the palate shows the position of the frontal section for panels (**I**–**N**). The upper sections (**I**,**J**) are from the area indicated by the red line (**H**), the middle one (**K**,**L**) by blue line, and the lower ones (**M**,**N**) by the black line. (**I**–**N**) Histological sections at E17.0 revealed the failure in palatal fusion in the Runx1 mutant shelves (J,L, arrowheads). Scale bar: 200 μm. pp, primary palate; sp, secondary palate; ns, nasal septum; vo, vomeronasal organ; if, incisive foramen; 1St, 1st rugae; 2nd, 2nd rugae.

As presumed, the present *K14-Cre/Runx1*^fl/fl^ mice demonstrated an anterior cleft that was similar to that of previous *Gata1-Runx1*/*Runx1*^−/−^ mice and survived after birth. An anterior cleft palate was confirmed in the mutants at P50 (Figs 1A,B and S2), clearly indicating that anterior cleft is not due to delayed development of the palate. This palatal phenotype was evident at P0 in more than 90% of the mutants (Fig. 1C,D,G). The palatal phenotype was not evident at E15.0 by direct observation (Fig. 1E,F).

Histological analyses revealed that there was a cleft between the primary and secondary palate and in the first rugae area the secondary palate in *Runx1* mutants at E17.0 (Fig. 1I–L). In the more posterior regions, the fused palate in *Runx1* mutants did not make contact with the nasal septum, even though the septal cartilage was almost normal in size (Fig. 1K,L). Palatal phenotypes were not evident more posteriorly than the point of the 2nd rugae (Fig. 1M,N). Although we used different genetically modified mice to study the function of Runx1 in palatogenesis in this study, the current histological findings are similar to the previous ones^5^. These findings confirmed that Runx1 is essential in anterior-specific palatogenesis.

### Characterization of the fusing epithelium of Runx1 mutants

**Figure 2:**
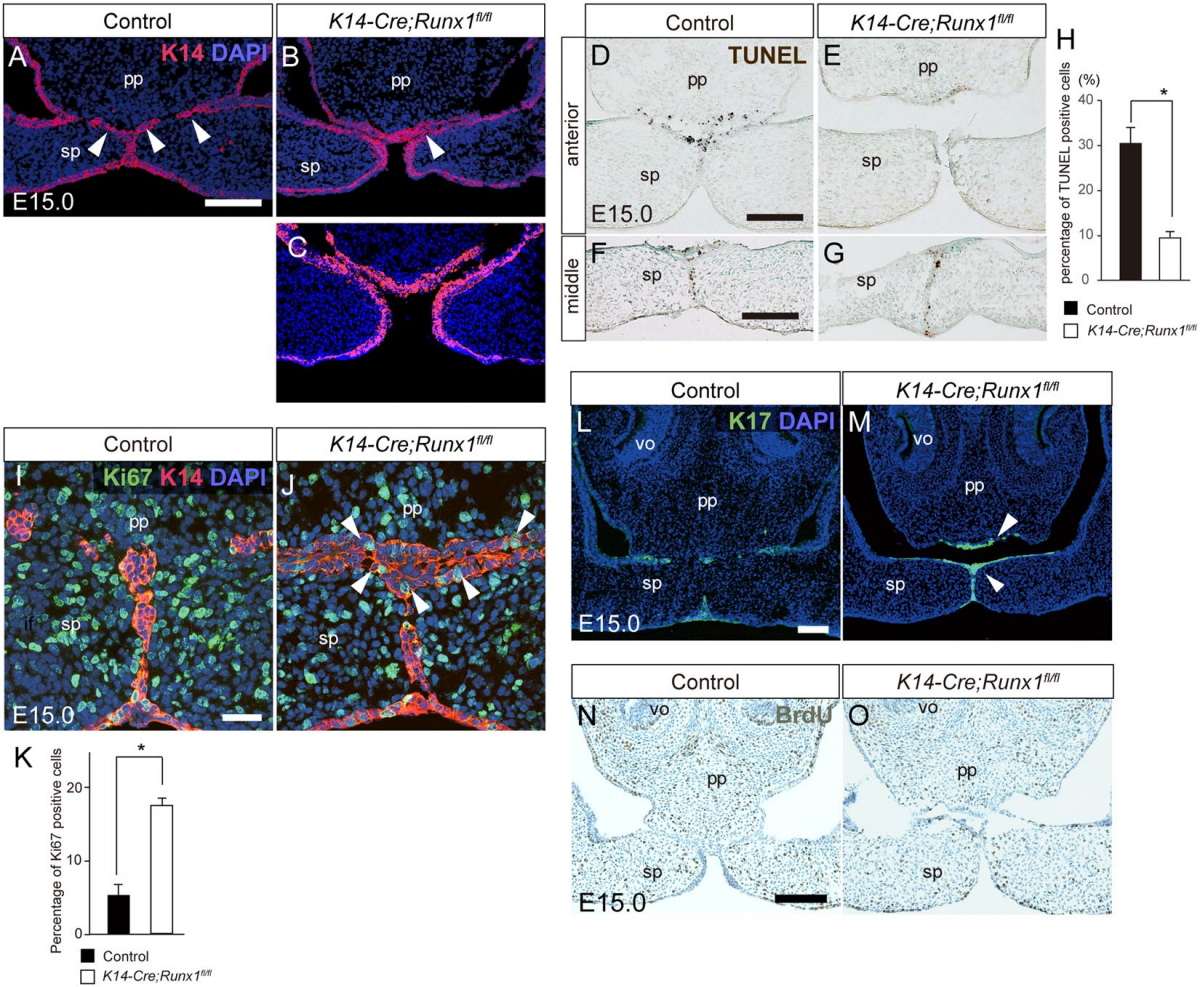
Palatal phenotypes of *K14-Cre/Runx1*^*fl/fl*^ mice. (**A**–**C**) Immunostaining for K14 at the boundary between the primary and secondary palate. (**A**) In controls, the epithelial remnants (arrowheads) are formed in the process of anterior palatal fusion. (**B**) In Runx mutants, 75% had partial contact at E15.0, and K14-immunoreactive epithelial remnants (B, arrowhead) were retained between the primary and the secondary palate: (**C**) 25% of the mutants did not have any contact. Scale bar: 100 μm. (**D**–**G**) TUNEL staining of frontal sections of the primary and secondary palates at E15.0. Scale bar: 100 μm. TUNEL-positive cells were fewer and sparse on the unfused epithelium in *Runx1* mutants. (**H**) The percentage of TUNEL-positive cells on the contacting epithelium. (**I**,**J**) Immunostaining for Ki67(green) and K14(red) revealed less proliferative cells in the epithelial remnants in wild-type mice (**I**), while some Ki67 signals were retained in the epithelial remnants in *Runx1* mutants (arrowheads in J). Scale bar: 50 μm. (**K**) The percentage of Ki67 positive cells. (**L**,**M**) Immunostaining for K17 (green) revealed that K17-immunoreactive periderm cells were retained on the surface of the nasal septum and the nasal side of the secondary palate in *Runx1* mutants. Nuclei were counterstained with DAPI (blue). The arrowhead indicates persistent periderm (arrowheads in M). Scale bar: 100 μm. (**N**,**O**) BrdU staining revealed that there were no marked changes in the BrdU signals in the palatal mesenchyme due to *Runx1* deficiency at E15.0. Scale bar: 200 μm. pp, primary palate; sp, secondary palate; ns, nasal septum; vo, vomeronasal organ.

Like the medial edge epithelium (MEE) that is localized at the junction between the secondary palates, the epithelial remnants appeared at the junction between the primary and the secondary palate at the anterior part of the palate (arrowheads in Fig. 2A). In *Runx1* mutant samples, immunostaining for K14 revealed that 75% of the mutants (15/20) at E15.0 had partial contact between the primary and secondary palate, and that thick K14-immunoreactive epithelial remnants (arrowhead in Fig. 2B) were detected at the interface. In contrast, 25% of the mutants (5/20) showed no contact between the nasal septum and the secondary palate and epithelial remnants were not formed at E15.0 (Fig. 2C). It has been established that, during epithelial fusion, the MEE ceases proliferation and undergoes apoptosis^22,23^, and the periderm overlying the fusing epithelium is removed^24^. Thereafter, the epithelial remnants need to degrade to achieve mesenchymal confluence^25^.

In this study, we first performed *in vivo* characterization of these anterior epithelial remnants. TUNEL staining revealed evident apoptosis in controls (Fig. 2D), whereas there were far fewer TUNEL-positive cells on the unfused epithelium of the *Runx1* mutants in the corresponding regions (Fig. 2E,H). In more posterior regions, the appearance of TUNEL-positive cells was not affected by *Runx1* deficiency (Fig. 2F,G).

The proliferative activity in the palatal epithelium of the *Runx1* mutant was also evaluated using Ki67 staining. In *Runx1* mutants, the thick epithelial remnants formed at the interface without any mesenchymal confluence due to partial contact between the nasal septum and the secondary palate. Double-staining for Ki67 and K14 showed that Ki67-immunoreactive proliferating cells were retained in these epithelial remnants in *Runx1* mutant, whereas Ki67-immunoreactive proliferating cells were less present at the fused epithelium in wild-types (Fig. 2I–K). Prior to fusion between the primary and the secondary palate at E14.5, Ki67-immunoreactive proliferating epithelium was sparsely present in the contacting palatal epithelium both in the control and the Runx1 mutant mice (Fig. S3A,B). Significant difference was not detected in the percentage of Ki67 positive cells between the control and the mutants (Fig. S3C).

During palatogenesis of the secondary palate, the periderm transiently forms a single flattened layer against premature adhesion of the fusing epithelium overlying the palatal shelf and is sloughed from the palatal surface in order to facilitate adherence and form a palatal seam^24^. In wild-type mice at E15.0, immunoreactivity to Keratin 17(K17), a marker of the periderm^26^, was sparsely detected in the epithelial remnants in the 2nd rugae area (Fig. 2L). In contrast, in *Runx1* mutants, K17-immunoreactive periderm cells were retained on the surface of the nasal septum, the primary palate and the the secondary palate (Fig. 2M). In the more anterior region corresponding to the 1st rugae, the unfused palatal process of the *Runx1* mutants was covered with K17-immunoreactive periderm, whereas K17-immunoreactive periderm was degraded in the control (Fig. S3D,E). Prior to fusion between the primary and the secondary palate at E14.5, K17-immunoreactive periderm covered the whole epithelial surface of the contacting palatal process both in the control and the *Runx1* mutants (Fig. S3D,E) These findings indicated that the periderm was not adequately removed in the primary palate and in the anterior-most region of the secondary palate, which is the corresponding to cleft regions in *Runx1* mutants.

At E17.0, although the nasal septal cartilage was normal in size in Runx1 mutants, a mesenchymal defect was not evident at the tissue overlying the vomeronasal organ (vo) (Fig. 1L). Therefore, insufficient growth of the nasal septum tissue may not have contributed to the cleft phenotypes.

BrdU staining revealed that there were no marked changes in the BrdU signals in the palatal mesenchyme due to *Runx1* deficiency at E15.0 (Fig. 2N,O). We also evaluated the vertical distance between the vomeronasal organ(vo) and the lower most surface of the primary palate and found that no significant differences were detected between the control and the Runx1 mutants (Fig. S4). It is therefore likely that the tissue defect observed at E17.0 was due to the secondary effect of contact failure between the secondary palate and the nasal septum, and not to growth failure of the nasal septum.

Taken together, these findings show that *Runx1* deficiency resulted in failure in the disintegration of the epithelial remnants of the anterior palate with retained proliferative activity, suppressed apoptosis, and inadequate periderm removal specifically at the junction between the primary and the secondary palates and at the anterior-most part of the secondary palate. These are novel findings indicating that Runx1 is involved in the disintegration of the fusing epithelium in morphogenesis.

### Downstream target of Runx1 signaling

**Figure 3:**
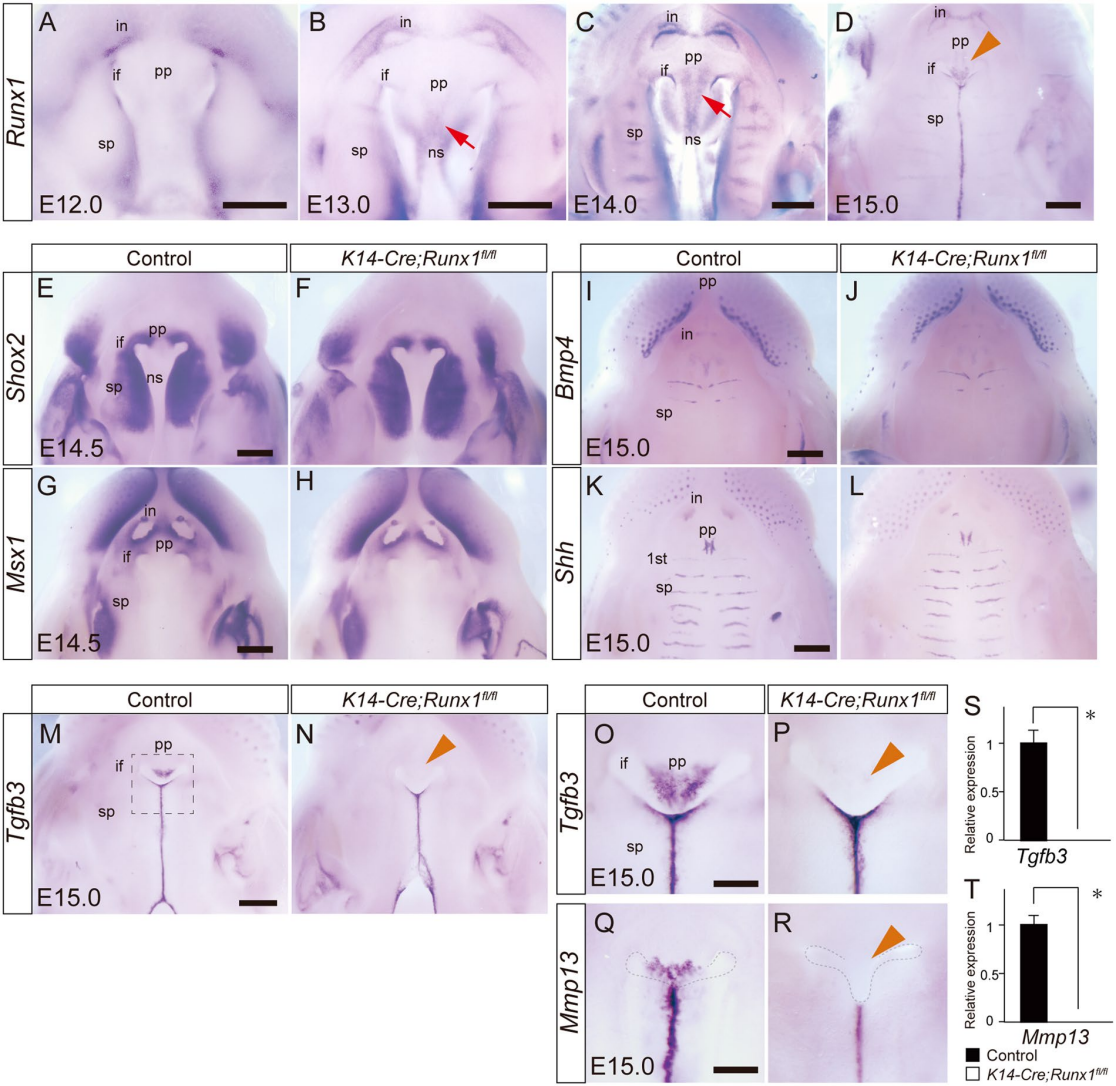
Downstream target molecules in Runx1 signaling in palatogenesis. (**A**–**D**) Whole-mount *in situ* hybridization analyses of *Runx1* in the developing palate of wild-type mice. *Runx1* is expressed at the fusing and fused epithelium of the secondary palate, the primary palate, and the nasal septum. Scale bar: 1000 μm. (**E**–**L**) Whole-mount *in situ* hybridization analyses showed that *Shox2*, *Msx1*, *Bmp4*, and *Shh* expression was not affected by *Runx1* deficiency. Scale bar: 500 μm. (**M**,**N**) The *Tgfb3* expression was markedly disturbed at the primary palate regions in K14-Cre/*Runx1*^*fl/fl*^ mice (yellow arrowhead). *Tgfb3* expression at the fused and/or fusing epithelium of the secondary palate was not affected by *Runx1* deficiency. Scale bar: 1000 μm. (**O**–**R**) Higher magnification of *Tgfb3* (inset of panel M) and *Mmp13* expression at the boundary between the primary and the secondary palate. The *Mmp13* expression was also markedly disturbed at the primary palate regions and at the first rugae area of the secondary palate in *Runx1* mutants. The yellow arrow indicates the region where *Tgfb3* and *Mmp13* expression was disturbed. (**S**,**T**) A qPCR analysis confirmed the marked downregulation of *Tgfb3* and *Mmp13* expression in *Runx1* mutants. Scale bar: 500 μm. Error bars, *p < 0.05; pp, primary palate; sp, secondary palate; ns, nasal septum; if, incisive foramen; 1^st^, 1^st^ rugae. Scale bar: 500 μm.

We further investigated the target molecules that may explain the cleft in *Runx1* mutants. The previous study demonstrated that *Runx1* is expressed in the fusing epithelium^5,27^. A whole-mount *in situ* hybridization analysis at E12.0, E13.0, E14.0 and E15.0 demonstrated that *Runx1* transcripts were detected at the primary palate from E13.0 and that *Runx1* was not specific to the anterior regions of the palate but widely expressed in the AP axis (Fig. 3A–D). In the primary palate regions, *Runx1* transcripts were abundant in the triangle regions delimited between the bilateral incisive foramen in the primary palate (arrowhead in Fig. 3D). The Runx1 expression in the primary palate and nasal septum was evident from E13.0 (arrows in Fig. 3B,C). *Runx1* transcripts were also specifically evident at the fusing surface of the secondary palate (Fig. 3D).

We next evaluated the possible downstream target molecules of Runx1 signaling in anterior palatogenesis. Anterior clefting is a rare phenotype in genetically manipulated mice, and several transcription factors and signaling molecules are recognized as anterior-specific^14,15,28,29^. *Shox2* is a homeobox gene expressed specifically in the anterior palate, and *Shox2* null mutant mice exhibit an anterior cleft palate^30^. *Msx1* is also an anterior-specific homeobox gene. Loss of *Msx1* expression leads to a complete cleft palate, and overexpression of BMP4 rescues this palatal phenotype. In this network, *Msx1* regulates *Bmp4* expression in the anterior mesenchyme, which subsequently induces *Shh* expression in the palatal epithelium^31^. The present whole-mount *in situ* hybridization revealed that the expression pattern of *Shox2*, *Msx2*, *Bmp4* or *Shh* did not deviate in *Runx1* mutants (Fig. 3E–L), indicating that Runx1 signaling is independent of the previously identified Msx1-Bmp4 and Shox2 signaling axes in anterior palatogenesis.

Among several signaling molecules, we found that *Tgfb3* was significantly decreased in *Runx1* mutants (Fig. 3M–P). Coinciding with anteriorly-specific palatal clefting in *Runx1* mutants, whole-mount *in situ* hybridization clearly demonstrated that downregulation of *Tgfb3* expression was specifically observed in the primary palate regions of *Runx1* mutants (arrowhead in Fig. 3N), while *Tgfb3* expression did not deviate in the secondary palate (Fig. 3M,N). A qPCR analysis of microdissected tissue confirmed such significant and spatially-specific downregulation in *Tgfb3* expression in the primary palate (Fig. 3S).

Whole-mount *in situ* hybridization also revealed that *Mmp13* was remarkably downregulated in the primary palate of *Runx1* mutants. Reduction was also evident at the anterior portion of the secondary palate anterior to the 2nd rugae (Fig. 3Q,R). A qPCR analysis also confirmed it (Fig. 3T).

*Mmp13* is a downstream target of *Tgfb3* during mammalian palatogenesis^32^, and spatial downregulation of *Mmp13* almost coincided with the spatial downregulation of *Tgfb3* at the primary palate. Whole-mount *in situ* hybridization analyses demonstrated that the distribution of *Mmp13* expression in the wild-types palate almost coincided with that of *Runx1* (Fig. 3D,Q).

Furthermore, as stated before, our *Runx1* mutants exhibited impaired removal of the periderm, which is similar to the epithelial phenotypes in *Tgfb3* null mutants^33,34^. Taken together, these findings indicated that *Runx1* deficiency results in the downregulation of *Tgfb3* expression specifically in the primary palate, and this Runx1-Tgfb3 signaling axis is a novel regulatory pathway that is independent of the Shh, Shox2 and Msx1-Bmp4 pathways.

**Figure 4:**
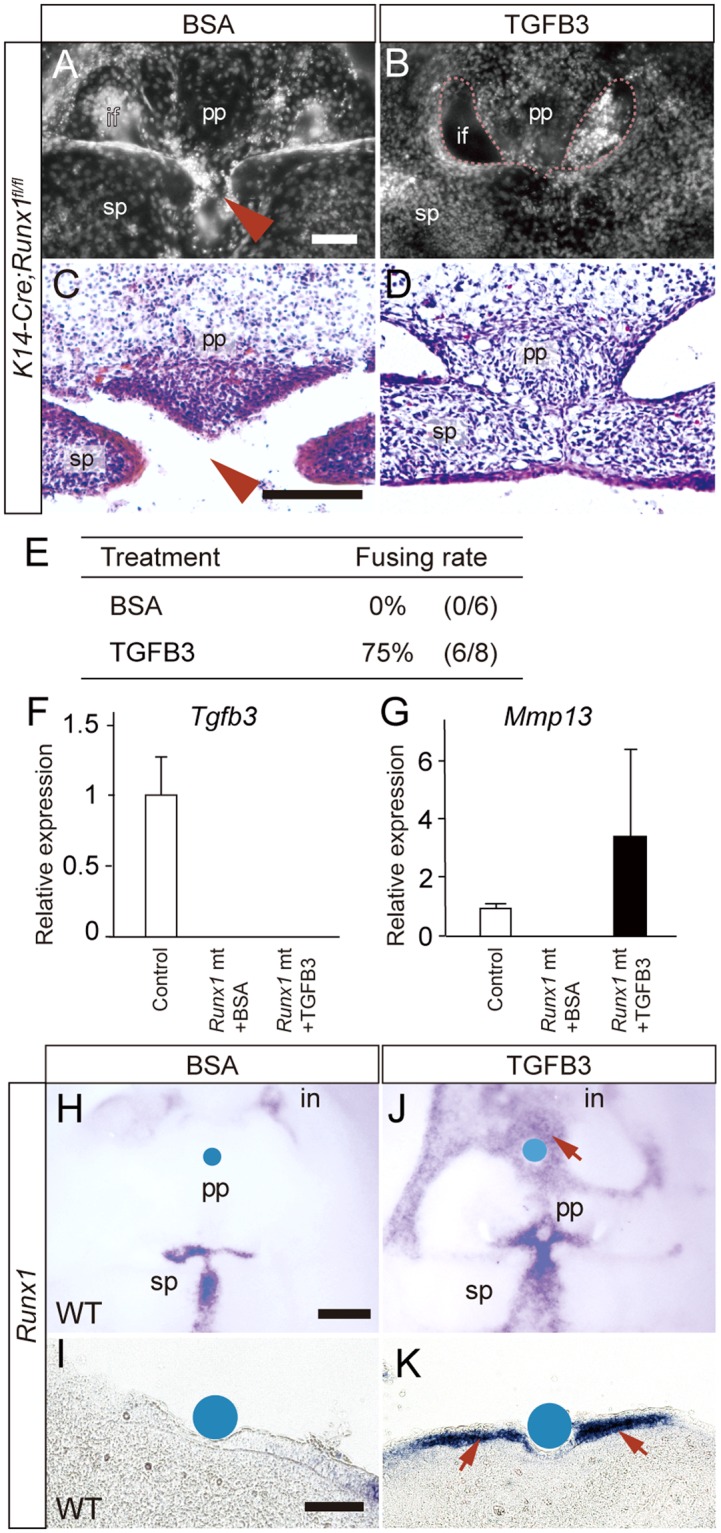
Rescue of cleft palate in Runx1 mutants by application of TGFB3. (**A**,**B**) Occlusal view of *Runx1* mutant palate cultured with BSA or TGFB3 protein beads. TGFB3 rescued the clefting of *Runx1* mutants (arrowhead). (**C**,**D**) A histological analysis confirmed the fusion by TGFB3 application. Scale bar: 100 μm. (**E**) The table shows the success rate of rescue. (**F**,**G**) A qPCR analysis of the rescued palatal tissues of *Runx1* mutants. The expression of *Tgfb3* and *Mmp13* was evaluated in the tissues of control and *Runx1* mutants with BSA or TGFB3 protein beads. (**H**–**K**) *In situ* hybridization analyses of protein-planted tissue. TGFB3-soaked beads induced the ectopic expression of *Runx1*. Control BSA beads failed to induce *Runx1*. Scale bar: 100 μm (upper), 200 μm (lower).

To further confirm the significance of the downregulation of *Tgfb3* signaling in *Runx1* mutants, we investigated whether or not treatment with TGFB3 beads can rescue the palatal clefting of *Runx1* mutants. TGFB3 protein beads or BSA-containing beads as controls were placed at the primary palate. After 48 h, the TGFB3 beads did indeed rescue the cleft palate formation (Fig. 4A,B). A histological analysis confirmed that the epithelial remnants between the primary and secondary palates and at the anterior-most part of the secondary palate were almost removed in order to allow for mesenchymal continuity (Fig. 4C,D). The success rate of rescuing the cleft palate was 75% (Fig. 4E). A qPCR analysis of microdissected tissue demonstrated that the TGFB3 beads induced *Mmp13* expression without upregulation of the *Tgfb3* mRNA expression (Fig. 4F,G). These TGFB3 rescue studies therefore confirmed that Tgfb3 is an essential and critical target molecule in Runx1 signaling in the anterior palatal fusion.

### Bidirectional Runx1-Tgfb3 signaling in the palate

We also evaluated whether or not Tgfb3 induces *Runx1* expression in the reverse direction in the palatal tissues. We found that TGFB3 protein beads induced ectopic *Runx1* expression in the palates in culture (Fig. 4H–K), indicating that Runx1-Tgfb3 signaling is reciprocally regulated.

### Stat3 activity in the Runx1-Tgfb3 axis

**Figure 5:**
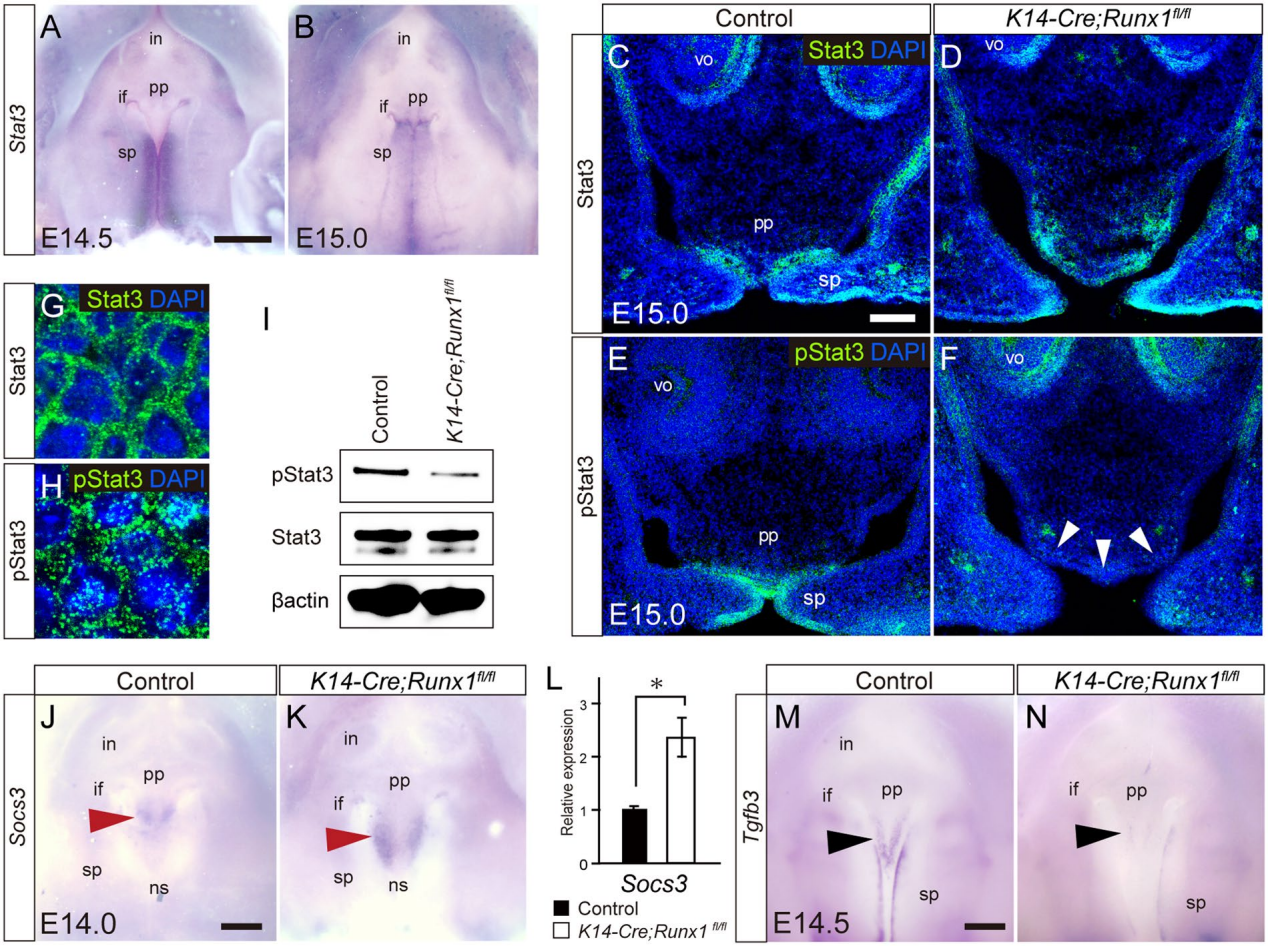
Stat3 activity in the palate of *Runx1* mutants. (**A**,**B**) Whole-mount *in situ* hybridization analyses of the expression of *Stat3* at E14.5 and E15.0 palate. Scale bar: 500 μm. (**C**–**F**) Immunofluorescence analyses of STAT3 (C,D, green) and phosphorylated STAT3 (E,F,green) of control and *K14-Cre/Runx1*^*fl/fl*^ mice. Nuclei were counterstained with DAPI (blue). The arrowhead indicates(I) decreased pStat3 immunoreactivity at the primary palate and at the first rugae area of the secondary palate of Run1 mutants. Scale bar: 100 μm. (**G**,**H**) Confocal microscopy confirmed that Stat3 immunoreactivity was detectable only in the cytoplasm, whereas phosphorylated Stat3 (pStat3) was detected in the perinuclear region and Stat proteins were translocated into the nucleus. (**I**) A Western blot analysis confirmed that pStat3 immunoreactivity was markedly downregulated by *Runx1* deficiency. (**J**,**K**) Whole-mount *in situ* hybridization analyses showed that *Socs3* mRNA was specifically localized in the primary palate at E14.0 (arrowhead) and that the *Socs3* expression was further upregulated by Runx1 deficiency (arrowhead). Scale bar: 200 μm. (**L**) qPCR analysis confirmed the significant upregulation of *Socs3* expression in *Runx1* mutants. *p < 0.05. (**M**,**N**) Whole-mount *in situ* hybridization analyses of *Tgfb3* in the unfused palate at E14.5. Scale bar: 200 μm. pp, primary palate; sp, secondary palate; ns, nasal septum; if, incisive foramen; in, incisor germ.

Runx1 acts is a tumor promoter in mouse skin cancer formation and maintenance by promoting Stat3 activation^16,17^ and *Tgfb3* expression is often upregulated in cancer formation^35^. We therefore evaluated the Stat3 activity in anterior palatal fusion in *Runx1* mutants. Whole-mount *in situ* hybridization analyses for *Stat3* mRNA demonstrated that the *Stat3* expression is particularly intense in the fusing palate; however, the *Stat3* mRNA expression was not anterior-specific (Fig. 5A,B). Stat3 activity was evaluated based on the state of phosphorylation using immunohistochemistry, and we performed immunohistochemical analyses for phosphorylated-Stat3 (pStat3) and Stat3.

Stat3 immunoreactivity was evident in the palatal epithelium, and some signals were detected in the mesenchyme (Fig. 5C); the distribution of Stat3 immunoreactivity did not deviate in *Runx1* mutant palate (Fig. 5D). In contrast, pStat3 immunoreactivity was evident in the fusing or fused epithelium of the wild-type palates (Fig. 5E). Notably, in *Runx1* mutant palate, immunoreactivity to pStat3 was significantly downregulated at the boundary between the primary and secondary palate and at the first rugae area of the palatal process, which are the corresponding cleft regions in *Runx1* mutants (Fig. 5F), whereas the immunoreactivity was unchanged in the secondary palate (Fig. S5).

In this observation of the fusing epithelium, confocal microscopy confirmed that Stat3 immunoreactivity was detectable only in the cytoplasm, whereas pStat3 was detected in the perinuclear region, indicating that phosphorylated Stat proteins translocate into the nucleus (Fig. 5G,H). A western blot analysis also demonstrated that pStat3 was significantly reduced in the *Runx1* mutant primary palate, while Stat3 was not affected (Fig. 5I). The scanned full blots are presented in Supplementary Fig. S6.

### Socs3 mRNA in Runx1 mutants

Then, in order to understand why pStat3 immunoreactivity was disturbed specifically in the nasal septum and the primary palate regions, we investigate the possible deviation of *Socs3* mRNA on *Runx1* deficiency. In cytokine signaling pathways, SOCS3 suppress Stat3 phosphorylation via interference with Jak2^36^. In keratinocytes, Runx1 binds to the *SOCS3* promoters to represses their transcription and ultimately upregulates Stat3 activity by enhancing phosphorylation due to *Runx1* depletion^9^. Hence, we hypothesized that Runx1 could regulate Stat3 activity specifically in the primary palate and the secondary palate at the at the first rugae regions through modulation of phosphorylation of Stat3 by downregulation of Socs3.

The present whole mount *in situ* hybridization demonstrated that *Socs3* mRNA expression was specifically localized on the surface of the nasal septum and the primary palate at E14.0 (Fig. 5J). The Intensity and the area of *Socs3* expression became further increased by Runx1 deficiency (Fig. 5K). qPCR analysis of these regions confirmed that *Socs3* mRNA expression was significantly increased in the *Runx1* mutant (Fig. 5L). Strikingly, the spatial distribution of *Socs3* mRNA expression in *Runx1* mutants almost overlapped with the regions where the *Tgfb3* expression was remarkably decreased in *Runx1* mutants (Fig. 5M,N). Collectively, *Runx1* deficiency leads to upregulated expression of *Socs3* specifically in the nasal septum and the primary palate, which might inhibit Stat3 activation in a spatially specific manner.

### Impairment of palatal fusion by Stat3 inhibitors

Given that spatiotemporal downregulation of *Tgfb3* expression overlaps with that of pStat3 immunoreactivity at the anterior palate, we hypothesized that Stat3 activity might directly or indirectly regulate *Tgfb3* expression, and concomitant impairment of *Tgfb3* induction might lead to the failure of palatal fusion. To evaluate the functional roles of Stat3 signaling, AG490, a selective Jak2/Stat3 inhibitor that prevents Stat3 phosphorylation, and S3I-201, a direct Stat3 inhibitor that blocks Stat3 dimerization and DNA-binding^37^, was applied in explanted palate at E15.0 for 48 h^38,39^.

**Figure 6:**
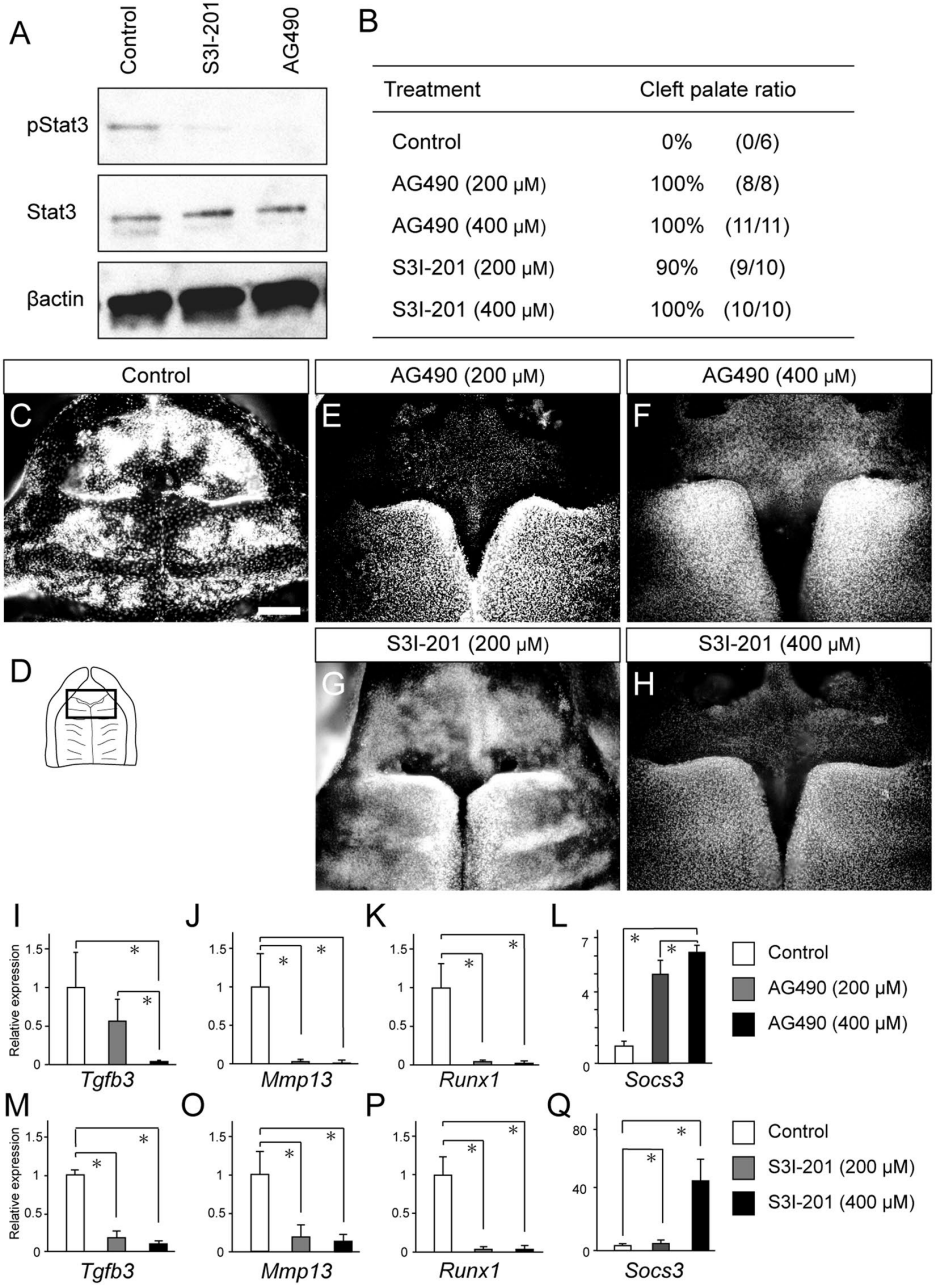
Application of Stat3 inhibitors leading to cleft palate in culture. (**A**) A Western blot analysis showed that the application of Stat3 inhibitors of Ag490 or S3I-201 suppressed immunoreactivity to pStat3 but not to Stat3. (**B**) The incidence of cleft palate by Stat3 inhibitors. (**C**–**H**) Occlusal views of the palates treated with Stat3 inhibitors. Both Ag490 and S3I-201 impaired the palatal fusion, while the BSA-treated control group showed complete fusion of the palate. The boxed region in the diagram was magnified to evaluate the cleft formation (**D**) if, incisal foramen. (**I**–**Q**) A qPCR analysis of the palatal tissues treated by AG490 or S3I-201. A qPCR analysis showed that application of Stat3 inhibitors disturbed the expression of *Tgfb3* and *Mmp13* and enhanced the *Socs3* expression in a dose-dependent manner.

A Western blot analysis confirmed that application of Stat3 inhibitors of Ag490 or S3I-201 almost suppressed immunoreactivity to pStat3, while Stat3 immunoreactivity was not affected (Fig. 6A). The scanned full blots are presented in Supplementary Fig. S7. In our *in vitro* culture system, the explanted palate fused by 100% in control mice (Fig. 6B,C). AG490 treated palate exhibited failure in fusion at both 200 and 400 µM by 100% (Fig. 6B,E,F). S3I-201 also impaired palatal fusion at 200 and 400 µM by almost 100% (Fig. 6B,G,H).

To further clarify the present hypothesis, a qPCR analysis of the microdissected primary palate revealed that the expression of *Tgfb3*, *Mmp13 and Runx1* was reduced significantly in a dose-dependent manner when pStat3 inhibitors were applied (Fig. 6I,J,K), whereas *Socs3* expression was upregulated in a dose-dependent manner (Fig. 6L). S3I-201 also downregulated *Tgfb3*, *Mmp13 and Runx* and upregulated *Socs3* significantly (Fig. 6M–Q).

These findings confirmed that the modification of Stat3 activity was able to influence the expression of *Tgfb3* directly or indirectly and subsequently regulate the anterior palatal fusion in culture.

## Discussion

*Runx1* deficiency results in anterior cleft palate between the primary and secondary palate and at the anterior-most region of the secondary palate. Using conditional *Runx1* null mutant mice, we showed that Tgfb3 is a critical downstream target in this pathogenesis and that the Runx1-Tgfb3 signaling axis is reciprocal and specific in the anterior regions of palatogenesis. In this process, we demonstrated that Stat3 mediates this Runx1-Tgfb3 signaling axis and that *Runx1* deficiency upregulates the expression of *Socs3*, an inhibitor of Stat3 signaling and specifically expressed in the primary palate regions. We further demonstrated that the pharmaceutical modification of Stat3 affects Runx1-Tgfb3 signaling and the palatal fusion *in vitro*.

In the present study, we found that Tgfb3 is a critical downstream target in *Runx1* mutants with anterior clefting and that the expression of *Shh*, *Shox2*, *Msx1*, or *Bmp4*, which are anterior-specific genes in palatogenesis^14^, was not affected in these mutants. *Runx1* deficiency resulted in marked downregulation of *Tgfb3* in the epithelium in the primary palate and the nasal septum; however, the expression of *Tgfb3* in the secondary palate was not affected. Tgfb3 has been established as a critical molecule that regulates epithelial fusion, and null mutant mice display a complete cleft palate^40^. In agreement with the animal phenotypes in *Tgfb3* null mutant mice, the case-parent triad study demonstrated that *TGFB3* variant was a potential genetic risk factor for increasing risk of non-syndromic CP^41^. However, when *Tgfb3*^33^ or its receptor^34,42^ is deleted under K14 epithelial promoter, clefting appears only in the anterior regions. In accordance with these previous findings in *Tgfb3* mutants, the cellular phenotypes in the present *Runx1* mutants resembled those of *Tgfb3* null mutants with sustained proliferation, disturbed apoptosis, and a retained periderm. Furthermore, TGFB3 rescued the cleft phenotypes among *Runx1* mutants in culture. Taken together, our findings indicated that Tgfb3 is a critical downstream target of Runx1 signaling specifically in the anterior palatogenesis and that this Runx1-Tgfb3 signaling axis is a novel regulatory pathway that is independent of previously known molecular networks involved in palatogenesis.

*Tgfb3* promoter does not contain Runx1 consensus sites within 1 kb of the transcription start site^43^. Therefore, some molecules mediate the downregulation of *Tgfb3* due to *Runx1* deficiency. In mammary gland involution, gland epithelium disappears due to lysosomal-mediated cell death^44^, as observed in the fusing palatal epithelium. Such cellular behaviors towards disintegration resemble those of the fusing epithelium of the palate^5^, and *Tgfb3* expression is upregulated in the involuting epithelium^45^. During involution, the induction of cell death is correlated with the phosphorylation of Stat3^44^, and the removal of *Stat3* resulted in the inhibition of cell death in a similar fashion to that seen in the *Tgfb3* null mutant mammary glands^46^, collectively indicating that the upregulation of *Tgfb3* expression with activated Stat3 phosphorylation regulates the cell death in mammary gland involution. Furthermore, Runx1 maintained Stat3 activity in the epidermis and in skin cancer cells^16,17^. Given these previous findings, we hypothesized that Stat3 might mediate the Runx1-Tgfb3 signaling pathway in palatal fusion.

The involvement of Stat3 in palatogenesis has not been previously investigated. We found that *Runx1* deficiency induces the inhibition of phosphorylation of Stat3 specifically in the corresponding cleft regions in the *Runx1* mutants, and such inhibition of Stat3 activity almost demonstrated spatiotemporal overlapping with that of *Tgfb3* expression. As two different Stat3 inhibitors with different modes of actions impaired the palatal fusion *in vitro* with remarkable inhibition of *Tgfb3* expression in the primary palate, the present findings suggested that Stat3 may be important in palatogenesis as a critical mediator of Runx1-Tgfb3 signaling. Interestingly, TGFB3 protein beads also reciprocally induced pStat3 and ectopic *Runx1* mRNA expression. Although whether such regulatory control is direct or indirect remains unclear, the present findings indicated that the Runx1-Stat3-Tgfb3 regulatory pathway orchestrates reciprocal control of genes required for palatal fusion in the anterior regions.

Anterior clefting is a rare palatal phenotype in the genetically modified mice, however, spatial distribution of *Runx1* mRNA do not account why the Stat3 activation and *Tgfb3* mRNA expression is disturbed only in the primary palate and the nasal septum. In human cancer cells, *Runx1* loss impairs tumor initiation and maintenance and the growth of oral, skin, and ovarian epithelial human cancer cells. In a previous study using mouse keratinocytes, chromatin immunoprecipitation followed by quantitative PCR (ChIP-qPCR) analysis demonstrated Runx1 binds to the promoters of *Socs3*, Stat3 inhibitors, to represses their transcription, and ultimately upregulates Stat3 activity by enhancing phosphorylation^9^. Conversely, *Runx1* deficiency results in upregulation of *Socs3* expression and Stat3 inactivation. Our whole mount *in situ* hybridization analyses demonstrated that *Socs3* expression was evident specifically in the fusing epithelium of the nasal septum and the primary palate of wild-type and this *Socs3* mRNA was further upregulated by *Runx1* deficiency. Remarkably, spatial distribution of upregulated *Socs3* mRNA overlapped with the regions of downregulated pStat3 immunoreactivity and *Tgfb3* mRNA. Hence, involvement of Socs3 on Runx1-Tgfb3 signaling axis could, at least in part, account how *Runx1* deficiency leads to spatially-specific downregulation of Stat3 phosphorylation and *Tgfb3* mRNA in the primary palate and the nasal septum and at the anterior portion of the secondary palate, and subsequent anterior specific cleft palate.

A striking finding of our study is that the pharmaceutical application of two different types of Stat3 inhibitor disturbed the expression of *Runx1* and inhibited *Tgfb3* expression, leading to failure in palatal fusion in wild-type mice. Such downregulation of *Runx1* by Stat3 inhibitors was also supported by upregulated expression of *Socs3*, as observed in *Runx1* mutants. These findings showed that the Runx1-Tgfb3 signaling axis was affected by extrinsic modification of Stat3. It has been established that Stat3 senses an array of extracellular signals and rapidly responds to them by controlling the target gene expression^47^. Acute alcohol intake suppresses the Stat3 activity through excessive Socs3 activation in human monocytes^48^, and nicotine also induces anti-inflammatory action associated with Stat3 phosphorylation in peritoneal macrophages^49^. Although a previous study did not examine whether or not alcohol intake and/or smoking influence the Stat3 activity in palatal fusion, Stat3 might act as a point of convergence integrating extrinsic environmental input into the genetically defined intrinsic conditions, which might provide a novel understanding of the etiology and pathology of the cleft palate. In the pathogenesis of non-syndromic cleft palate, several genetic susceptibility factors and environmental risk factors have been identified, and a clinically relevant phenotype is identified only when a threshold effect is reached after combining each genetic and environmental susceptibility factor affecting each individual^1,2^. Prevention is the ultimate objective with regard to CP. The present findings have identified genetic targets that modify the environmental risk factors, and pharmaceutical modulation of Stat3 signaling may modify the Runx1-Tgfb3 signaling axis, suggesting its utility as a therapeutic procedure for preventing CP in patients with pathologic *TGFB3* variant.

One limitation associated with this study is that Stat3 signaling was inhibited by pharmacological Stat3 inhibitors. Given the possibility that Stat3 inhibitors also affect other signaling besides Stat3, we used two different Stat3 inhibitors with different mechanisms of action. In future experiments, we will generate epithelial-specific *Stat3* null mutants to directly confirm the function of Stat3. Previous studies using tissue-specific Stat3-deficient mice showed that Stat3 plays a crucial role in a variety of biological functions, including cell growth, anti-apoptosis, apoptosis and cell motility depending on the cell type and stimulus^9,19,47,50^. However, the cleft palate phenotype has not been reported using such *Stat3* mutant mice. In previous studies using epithelial-specific *stat3* deletion mice, *Keratin 5 (K5)* promoter was used for the epithelial-specific *Cre*-mediated deletion of the *Stat3* gene. *K14-Cre* is another *Cre* driver for generating epithelial-specific conditional null mutant mice, and interestingly, *Dicer*-null mutants driven by K14 and K5 do not exhibit similar appendage phenotypes. Indeed, *K14-Dicer* mutants exhibit more severe phenotypes at birth than *K5-Dicer* mutants, possibly due to the earlier onset of the *K14-Cre* driver^51^. Since palatal fusion occurs in the embryonic stages, it is likely that the *K14-Cre* driver is appropriate for evaluating the possible function of Stat3 in the fusing epithelium. We intend to investigate the palatal phenotypes using *K14-Cre* driven *Stat3* conditional null mice in future experiments.

**Figure 7:**
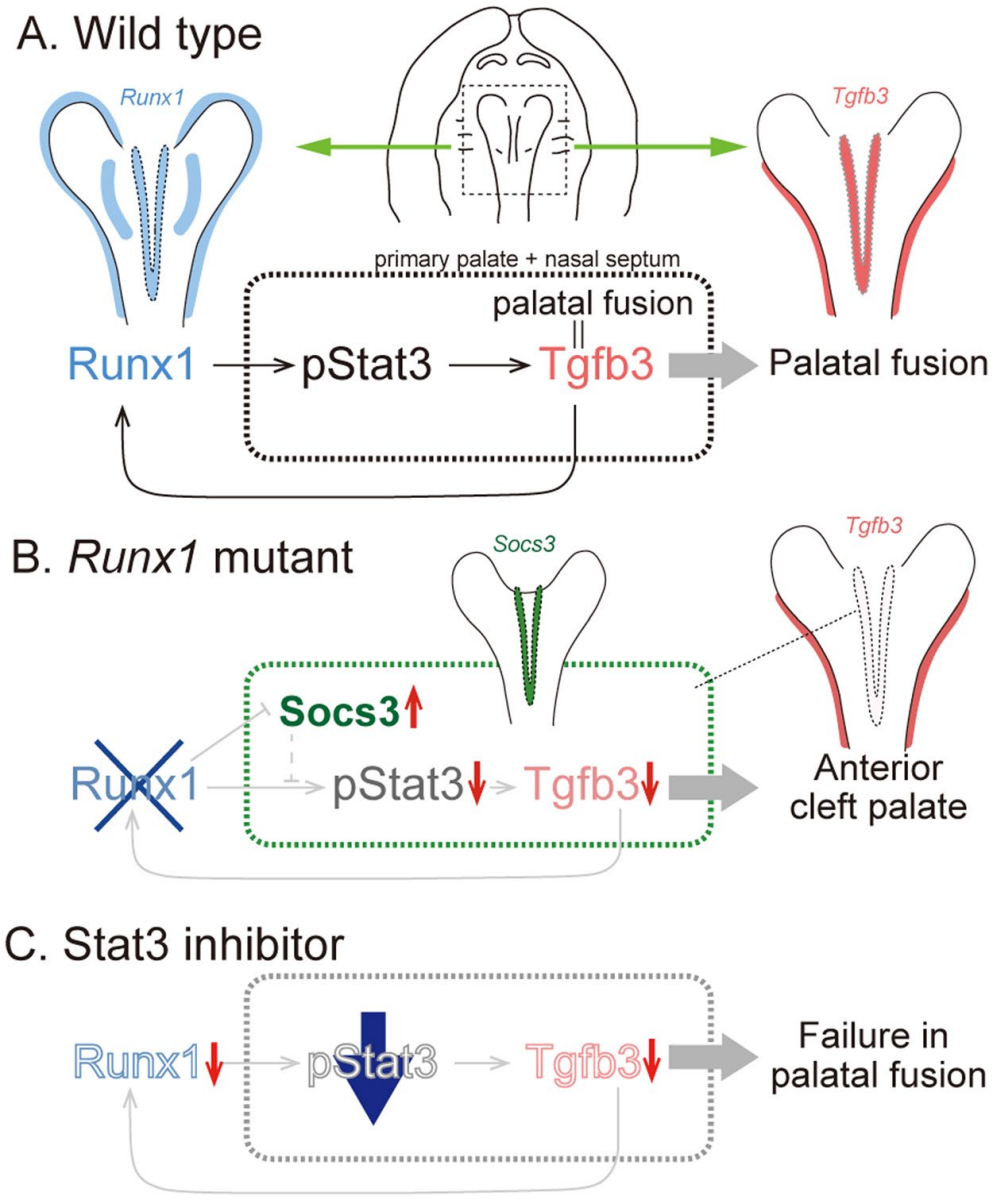
Runx1-Stat3-Tgfb3 signaling network regulate the fusion of the anterior palate. Schematic illustration of the key findings in this article. (**A**) Tgfb3 is a critical downstream target of Runx1 signaling, which regulate the palatal fusion between the primary and the secondary palate and at the anterior-most part of the secondary palate. The novel Runx1-Tgfb3 signaling axis is mediated by Stat3 phosphorylation. (**B**) *Tgfb3* is remarkably disturbed in *Runx1* mutants specifically in the primary palate and nasal septum with suppression of Stat3 phosphorylation. *Socs3* expression is localized in the primary palate and *Socs3* mRNA is upregulated by *Runx1* deficiency. (**C**) Stat3 inhibitor results in the failure of palatal fusion *in vitro* with significant downregulation of *Tgfb3* expression and Stat phosphorylation. Stat3 inhibitor further disturbs the *Runx1* expression.

In conclusion, we found that *Runx1* epithelial-specific deletion led to the failed disintegration of the contacting palatal epithelium and that Tgfb3 is a critical downstream target in the pathogenesis of anterior cleft palate in the mutants. This Runx1-Tgfb3 signaling axis is independent of previously known signaling systems and is reciprocally mediated by Stat3. In this process, expression of *Socs3*, an inhibitor of Stat3 signaling, was upregulated specifically in the primary palate by *Runx1* deficiency, which could explain, at least in part, how *Runx1* deficiency results in anteriorly specific clefting (Fig. 7A,B). Furthermore, the pharmaceutical application of Stat3 inhibitor disturbs the expression of *Runx1* as well as *Tgfb3* and leads to failure of palatal fusion in wild-type mice, suggesting that the Runx1-Tgfb3 signaling axis may be affected by extrinsic modification of Stat3 signaling (Fig. 7C). The involvement of Stat3 modification in Runx1-Tgfb3 signaling may offer novel insights into the physiologic and pathophysiologic regulation of the palatal fusion (Fig. 7A) and our study also clarifies potential therapeutic targets in the prevention and pharmaceutical intervention for cleft palate.

## Materials and Methods

### Animals

*Runx1*^−/−^ mice are lethal due to hemorrhaging at about E10.5 to E12.5, when the palatal development is not yet completed^5^. To determine the role of Runx1 in oral epithelium, we use epithelial-specific knockout mice created through the *Cre*/*loxP* system (*K14-Cre/Runx1*^*fl/fl*^). To generate *K14-Cre/Runx1*^*fl/fl*^ mice, we first mated heterozygous *K14-Cre* mice^52^ and *Runx1*^*fl/fl*^ mice^53^ to obtain *K14-Cre*/*Runx1*^*fl*/+^mice. These progenies were subsequently bred with *Runx1*^*fl/fl*^ mice. Genotyping was performed by the conventional polymerase chain reaction (PCR) method using each primer set to detect *Cre* (5′ CTCTGGTGTAGCTGATGATC 3′ and 5′ TAATCGCCATCTTCCAGCAG 3′) and the *loxP* site of *Runx1* (5′ GCGTTCCAAGTCAGTTGTAAGCC 3′ and 5′ CTGCATTTGTCCCTTGGTTGACG 3′). We used littermates that did not carry the *K14-Cre/Runx1*^*fl/fl*^ genotype as control animals. We also confirmed the efficiency of the *K14-Cre* recombination in anterior palatogenesis using Rosa26R reporter mice and X-gal staining, as shown in a previous study^54^.

### Assessment of palatal fusion and a histological analysis

The mouse embryonic heads were dissected in BGJb medium (Gibco). The palate was evaluated by direct observation and with a dissecting microscope. These tissue were fixed in 4% paraformaldehyde, equilibrated in graded sucrose, and embedded in Tissue-Tek (OCT compound, Sakura).

### Immunohistochemistry

Immunofluorescence staining was performed on 20-µm sections using polyclonal rabbit-anti-Ki67 (1:400, ab15580, Abcam), monoclonal rabbit anti-K17 (1:200, #4543, Cell Signaling Technology), monoclonal anti-K14 (1:200, ab7880, Abcam), monoclonal rabbit anti-phospho-Stat3 (pStat3, 1:200, #9145, Cell Signaling Technology), monoclonal rabbit anti-Stat3 (1:200, #9139, Cell Signaling Technology) overnight at 4 °C. Then, Alexa488-conjugated goat-anti-rabbit IgG (1:400, A21206, Molecular Probes) or Alexa546-conjugated goat-anti-mouse IgG (1:400, A11003, Molecular Probes) was used as secondary antibody. The sections were then counterstained with DAPI (1:500, Dojindo) and mounted with fluorescent mounting medium (Dako). At least three embryos of each genotype were used for each analysis.

### Laser microdissection

The mice embryonic maxilla were freshly embedded in OCT compound and frozen immediately. Tissues are serially sectioned at −20 °C on a cryostat (CM 1950, Leica) at a thickness of 25 μm. The maxilla was sectioned from anterior to posterior throughout anterior palate until the secondary palate appeared. The tissue sections were mounted and thawed on a film-coated slide. In total, there were 12–14 serial sections obtained from the anterior palate at E15.0 (section numbers varied due to the orientation of the frozen block). We stained these slides with cresyl violet dye staining. Anterior palate epithelial and mesenchymal tissue were dissected from the sections using a Leica Micro Laser System (LMD6500, Leica) and collected by tube.

### RNA extraction and real-time RT-PCR analyses

We used the laser-microdissected tissues of the control and *K14-Cre/Runx1*^*fl/fl*^ mice to extract total RNA. IsogenII (Nippon Gene) was used to extract total RNA according to the manufacturer’s protocol. Total RNA was reverse transcribed to cDNA using an oligo (dT) with avian myeloblastosis virus reverse transcriptase (Takara). For the real-time RT-PCR analysis, the cDNA was amplified with TaqDNA Polymerase (Toyobo) using a light cycler (Roche). The qPCR was carried out with *Gapdh* used as a housekeeping gene and analyzed as previously described^54^. Primer sequences are available in the Supplementary Fig. S8. At least three embryos of each genotype were used for each analysis.

### Whole-mount *in situ* hybridization

Whole-mount *in situ* hybridization was performed using fixed E12.0, E13.0, E14.0, E14.5 and E15.0 palates. The digoxigenin-labeled RNA probes used in this study were prepared using a DIG RNA labeling kit (Roche) according to the manufacturer’s protocol using each cDNA clone as the template. The probes were synthesized from fragments of *Runx1*, *Shox2*, *Msx1*, *Shh*, *Bmp4*, *Tgfb3*, *Socs3* and *Stat3* (Allen Institute for Brain Science) and were amplified with T7 and SP6 adaptor primers through PCR. After hybridization, the expression patterns for each mRNA were detected and visualized according to their immunoreactivity with anti-digoxigenin alkaline phosphatase-conjugated Fab fragments (Roche), as previously reported^2^. At least three embryos of each genotype were used for each analysis.

### TUNEL staining

TUNEL assay for apoptosis was conducted according to the manufacturer’s protocol (ApopTag; Chemicon). Frozen sections (10 μm) from samples were prepared. The sections were counterstained with methyl green. At least three embryos of each genotype were used for each analysis.

### *In vitro* culture of palatal shelves and rescue of the mutant cleft palate using TGFB3 protein

The palate was dissected and explanted from the E15.0 embryo and cultured on track-etched polycarbonate membrane filter (Nuclepore) in Trowell type organ culture with serumless, chemically defined BGJb medium (Gibco). In our dissection, the primary palate and the nasal septum were not excluded from our culture system. Affi-Gel beads (Bio-Rad) were incubated in TGFB3 (100 ng/μl, R&D Systems). Bovine serum albumin (BSA; Sigma-Aldrich) was used instead of recombinant protein for the control beads. The beads were immersed in recombinant protein or BSA at 37 °C for 60 min and placed on the primary palate of the explants using a pipette tube. After culture, the *in vitro* explants were fixed at each stage in 4% paraformaldehyde overnight and then processed for histological examination and qPCR analyses.

### Whole-head roller culture and treatment of Stat3 inhibitor

Embryo heads from E14.0 ICR mouse embryos were collected in BGJB, and the mandibles, tongues, and brains were removed. The remaining palatal tissue, including both the primary and secondary palate and the nasal septum, were cultured for 24–48 h in a whole-embryo culture incubator (RKI Ikemoto) at 37 °C. Palatal tissues were incubated in BGJb medium with or without AG490 (200–400 µM; Sigma-Aldrich) or STAT3 Inhibitor VI, S3I-201 (200–400 µM; Sigma-Aldrich). Tissues were harvested after 24 h of culture and processed for qPCR analysis.

### Western blot analysis

The dissected palatal tissues were lysed with RIPA buffer (nacalai tesque) supplemented with protease and phosphatase inhibitors (nacalai tesque). The lysates were centrifuged and the supernates were heated in denaturing Laemmli buffer (Bio-rad Laboratories). Proteins were separated by SDS-PAGE and transferred to Polyvinylidene difluoride membranes (Bio-rad Laboratories). The membranes were incubated with either anti-Stat3 (1:1000, #9139, Cell Signaling Technology), anti-pStat3 (1:1000, #9145, Cell Signaling Technology) and beta-actin (1:2000, Sigma). The bound antibodies were detected with HRP-linked antibody (1:1,000, Cell Signaling Technology) and the ECL detection kit (Bio-rad Laboratories).

### Statistical analyses

Quantitative variables in two groups were compared using the Mann-Whitney *U* test. Differences among the three groups were determined using the analysis of variance (ANOVA) test, and significant effects indicated by the ANOVA were further analyzed with post hoc Bonferroni correction. *P* values < 0.05 were considered significant. Significance was determined using the statistical analysis software program JMP, version 5 (SAS Institute Inc.).

### Study approval

All animal experiments were carried out in strict accordance with the recommendations in the Guide for the Care and Use Committee of the Osaka University Graduate School of Dentistry, Osaka, Japan. The protocol was approved by the Committee on the Ethics of Animal Experiments of Osaka University Graduate School of Dentistry. Mice were housed in the animal facility at the Department of Dentistry, Osaka University. Welfare guidelines and procedures were performed with the approval of the Osaka University Graduate School of Dentistry Animal Committee.

## Electronic supplementary material

Supplementary Figures
