# Supplementary material for: Runx1-Stat3-Tgfb3 signaling network regulating the anterior palatal development
Source: Sci Rep. 2018 Jul 25;8:11208. doi: 10.1038/s41598-018-29681-3 (PMC6060112; doi:10.1038/s41598-018-29681-3)
Supplement: Supplementary file 1 — Supplementary Figures [file 41598_2018_29681_MOESM1_ESM.pdf]

Runx1-Stat3-Tgfb3 signaling network regulating the anterior palatal development

Safiye E. Sarper[1], Hiroshi Kurosaka[1], Toshio Inubushi[1], Hitomi Ono[2], Koh-ichi Kuremoto[3], Takayoshi Sakai[2], Ichiro Taniuchi[4], Takashi Yamashiro[1]*.

[1]Department of Orthodontics and Dentofacial Orthopedics, Osaka University Graduate School of Dentistry, Osaka, Japan

[2]Department of Oral-facial Disorders, Osaka University Graduate School of Dentistry, Osaka, Japan

[3]Department of Advanced Prosthodontics, Graduate School of Biomedical & Health Sciences, Hiroshima University, Hiroshima, Japan

[4]Laboratory for Transcriptional Regulation, RIKEN Research Center for Allergy and Immunology, Yokohama, Japan

Supplementary Figure S1

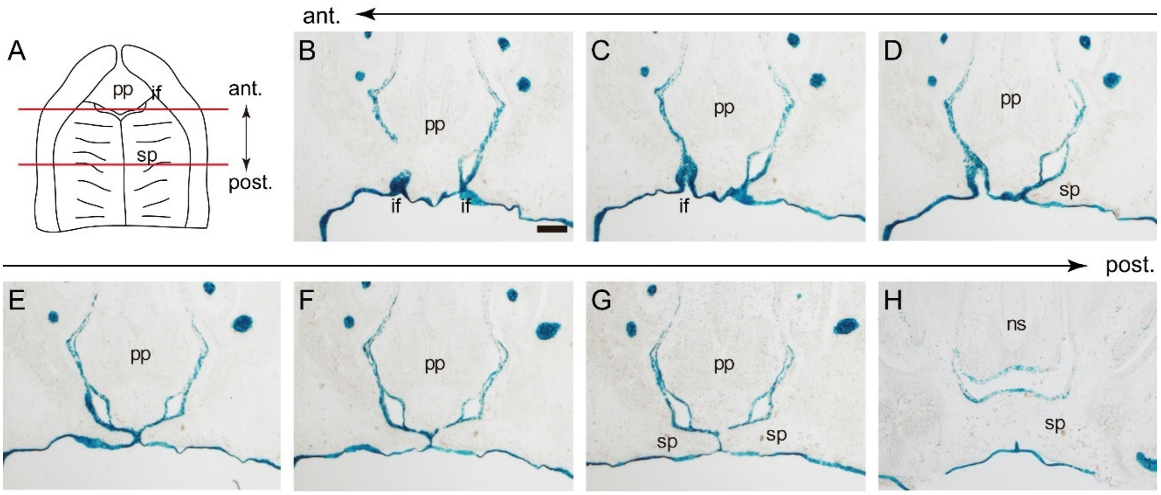

Supplemental Figure S1. β-gal staining pattern at the boundary between the primary and the secondary palate from *K14-Cre;R26R* embryos. (A) A diagram of the occlusal view of the palate shows the position of the serial frontal section for panels (B-H). (B-H) β-gal-positive cells were intense at the epithelium overlying the palatal process of the secondary palate and the primary palate at E15.0. The positive cells were also evident in the contacting (B-E) and fused epithelium (F-H); however, no positive cells were detected in the mesenchyme underlying the palatal epithelium. Bar=200 μm. pp, primary palate; sp, secondary palate; if, incisive foramen; ns, nasal septum.

Supplementary Figure S2

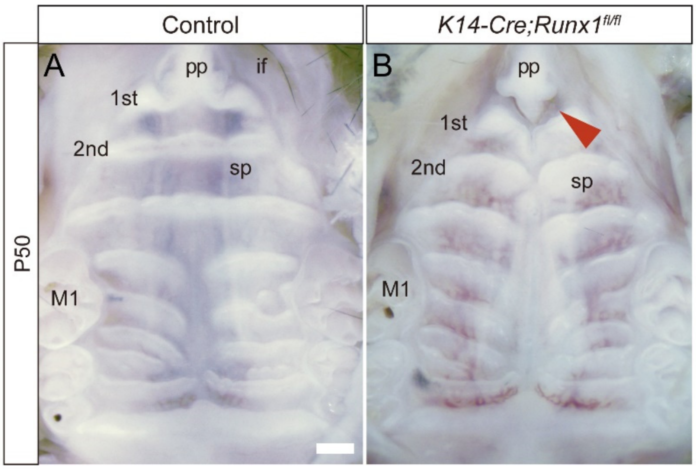

Supplementary Figure S2. Palatal phenotypes of *Runx1* mutants. (A,B) Occlusal views of the palate at P50. Anterior cleft is evident in *Runx1* mutants. Scale bar: 1 mm. pp, primary palate; sp, secondary palate; if, incisive foramen; 1st, 1st rugae; 2nd, 2nd rugae; M1, the 1st molar.

Supplementary Figure S3

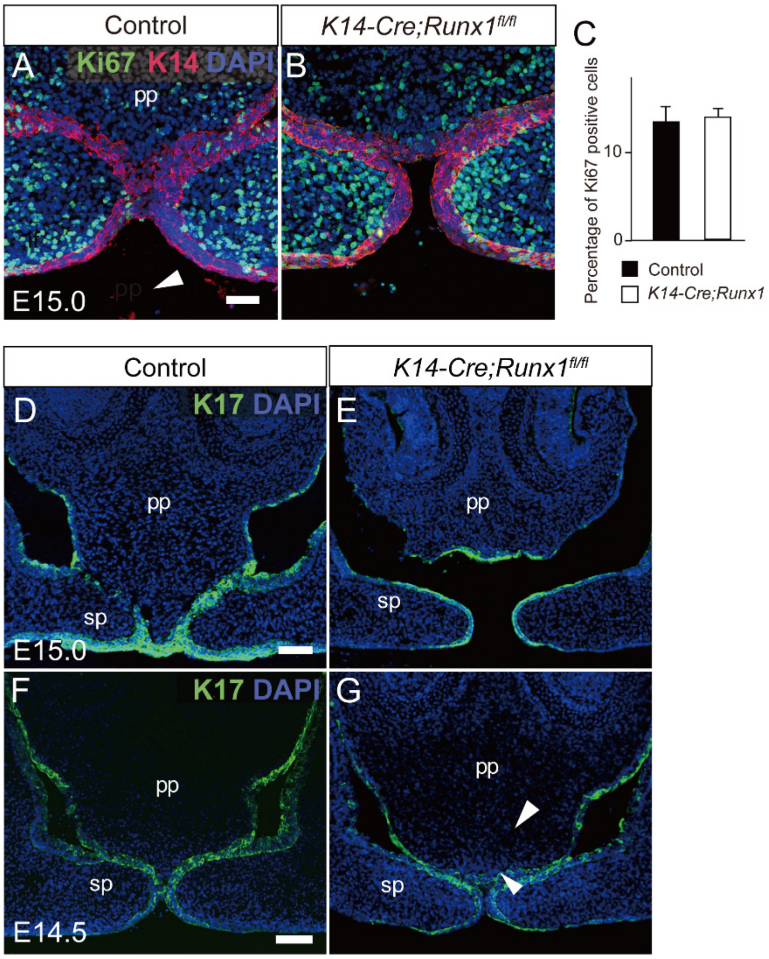

Supplemental Figure S3. Palatal phenotypes of *K14-Cre/Runx1*$^{fl/fl}$ mice. (A,B) Immunostaining for Ki67(green) and K14(red) revealed that proliferative cells in contacting but unfused palatal epithelium of the secondary palatal process and the primary palate were visible in the control (A) and Runx1 mutant (B) mice at E14.5. Scale bar: 50 μm. (C) Significant difference was not detected in the percentage of Ki67 positive cells between control and Runx1 mutants. (D,E) Immunostaining for K17 (green) in the 1st rugae region of the palate at E15.0. The unfused palatal process of the *Runx1* mutants was covered with K17-immunoreactive periderm (E), whereas K17-immunoreactive periderm was degraded in the control (D). (F,G) At E14.5, K17-immunoreactive periderm cells (arrowheads) covered almost the whole surface of contacting palatal process and both in the control (F) and Runx1 mutant (G) mice at E14.5. Scale bar: 100 μm. Nuclei were counterstained with DAPI (blue).

Supplementary Figure S4

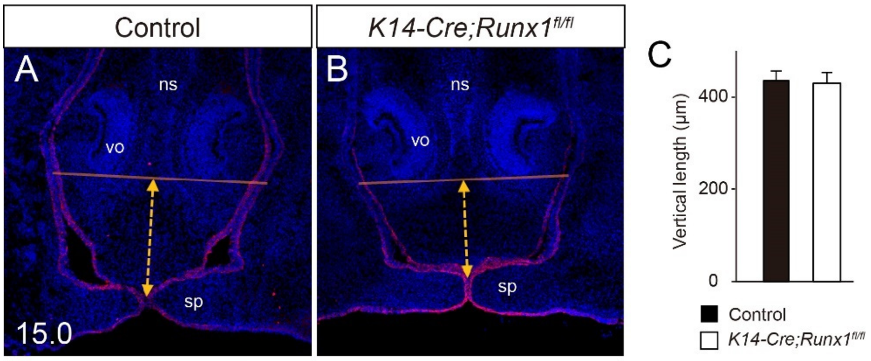

Supplementary Figure S4. The vertical growth of the anterior palate. (A,B) On the frontal section, the vertical distances between the vomeronasal organ(vo) and the lower-most surface of the primary palate of the control (A) and Runx1 mutants (B) was compared. (C) Significant differences were not observed. sp, secondary palate; ns, nasal septum; vo, vomeronasal organ.

Supplementary Figure S5

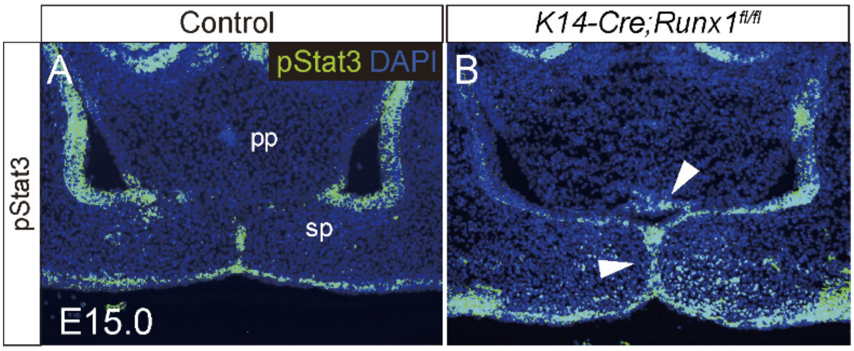

Supplemental Figure S5. Stat3 activity in the palate of *Runx1* mutants. (A,B) Immunofluorescence analyses of phosphorylated STAT3 of control (A) and *K14-Cre/Runx1*$^{fl/fl}$ (B) mice. Nuclei were counterstained with DAPI (blue). pStat3 immunoreactivity was evident in the fusing epithelium of the primary palate and the secondary palate in the 2nd rugae region both in control and Runx1 mutants. Scale bar: 100 µm.

Supplementary Figure S6

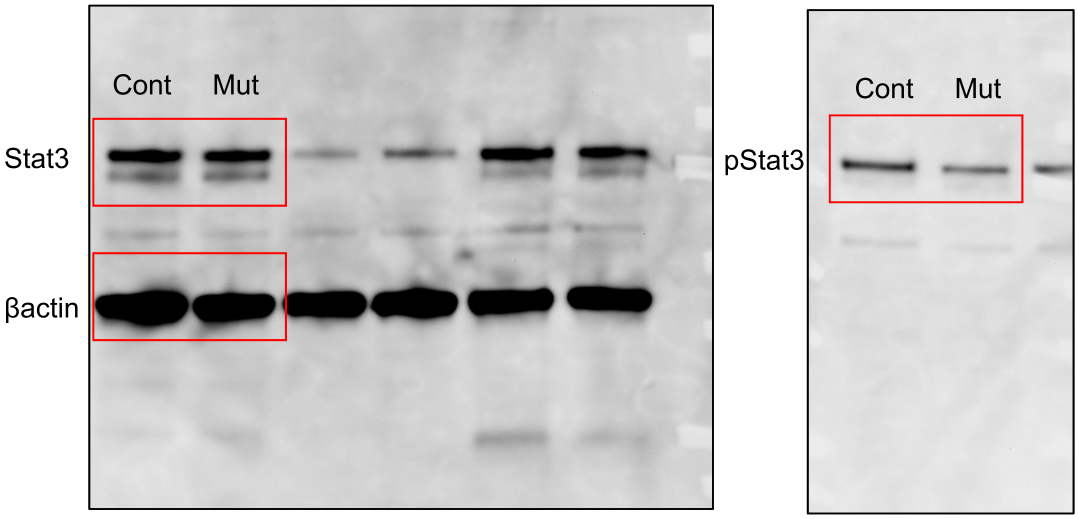

Supplementary Figure S6. Full-length blots of pStat3, Stat3 and βactin in the primary palatal tissue of control and Runx1 mutants (shown as cropped images in Figure 5).

Supplementary Figure S7

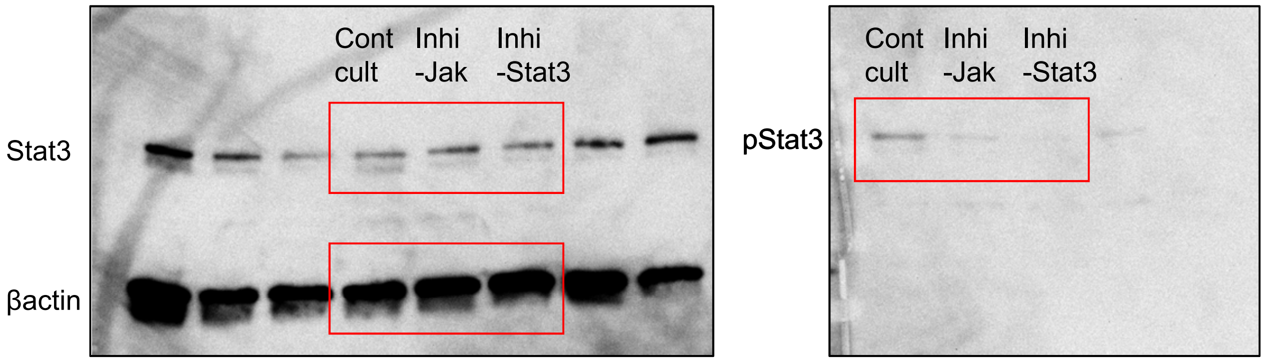

Supplementary Figure S7. Full-length blots of pStat3, Stat3 and βactin in the primary palatal tissue with or without Stat3 inhibitors (shown as cropped images in Figure 6).

Supplementary Figure S8

Primers for *in situ* hybridization:

Runx1 fwd: 5´-GTTCAAGCCTGGAAGTCCTG-3´

Runx1 rev: 5´-TTAGTTGTCCTTCTTGATGCGG-3´

Tgfb3 fwd: 5´-CGCCCCTGCCAGAATGGTG-3´

Tgfb3 rev: 5´-GACTTGACCTTGGCCGAC-3´

Mmp13 fwd: 5´-GGCTAATGACTGGCTGACC-3´

Mmp13 rev: 5´-TTAGACCTTGGCCTGCTCCA-3´

Socs3 fwd: 5´-CAAGAACCTACGCATCCAGTG-3´

Socs3 fwd: 5´-CCAGCTTGAGTACACAGTCGAA-3´

Supplementary Figure S8. Primers for quantitative PCR.
